# Characterization of Ly108-H1 Signaling Reveals Ly108-3 Expression and Additional Strain-Specific Differences in Lupus Prone Mice

**DOI:** 10.3390/ijms24055024

**Published:** 2023-03-06

**Authors:** Svend Rietdijk, Marton Keszei, Wilson Castro, Cox Terhorst, Ana C. Abadía-Molina

**Affiliations:** 1 Unidad de Inmunología, IBIMER, CIBM, Universidad de Granada, 18016 Granada, Spain; 2 Division of Immunology, Beth Israel Deaconess Medical Center, Harvard Medical School, Boston, MA 02215, USA; 3 Department of Gastroenterology and Hepatology, OLVG Hospital, 1091 AC Amsterdam, The Netherlands; 4 Departamento de Bioquímica y Biología Molecular III e Inmunología, Facultad de Medicina, Universidad de Granada, 18016 Granada, Spain

**Keywords:** Ly108, NTB-A, SLAMF6, isoforms, SAP, SLAM

## Abstract

Ly108 (SLAMF6) is a homophilic cell surface molecule that binds SLAM-associated protein (SAP), an intracellular adapter protein that modulates humoral immune responses. Furthermore, Ly108 is crucial for the development of natural killer T (NKT) cells and CTL cytotoxicity. Significant attention has been paid towards expression and function of Ly108 since multiple isoforms were identified, i.e., Ly108-1, Ly108-2, Ly108-3, and Ly108-H1, some of which are differentially expressed in several mouse strains. Surprisingly, Ly108-H1 appeared to protect against disease in a congenic mouse model of Lupus. Here, we use cell lines to further define Ly108-H1 function in comparison with other isoforms. We show that Ly108-H1 inhibits IL-2 production while having little effect upon cell death. With a refined method, we could detect phosphorylation of Ly108-H1 and show that SAP binding is retained. We propose that Ly108-H1 may regulate signaling at two levels by retaining the capability to bind its extracellular as well as intracellular ligands, possibly inhibiting downstream pathways. In addition, we detected Ly108-3 in primary cells and show that this isoform is also differentially expressed between mouse strains. The presence of additional binding motifs and a non-synonymous SNP in *Ly108-3* further extends the diversity between murine strains. This work highlights the importance of isoform awareness, as inherent homology can present a challenge when interpreting mRNA and protein expression data, especially as alternatively splicing potentially affects function.

## 1. Introduction

The SLAM family (SLAMF) of transmembrane protein receptors are involved in many important immune processes [1,2,3,4,5]. Most members of the SLAMF are self-ligands on the cell surface and have immunoreceptor tyrosine-based switch motifs (ITSM) in their cytoplasmic tails enabling them to bind SAP as well as EAT-2, ERT, SHP-1, and SHIP-1 [6,7,8]. SAP deficiency in humans results in the often-fatal X-linked lymphoproliferative disease (XLP) that usually manifests as an inability to control EBV infections and the resulting inflammation [9,10,11,12]. SAP is also critical for the generation of humoral immunity as well as for the development of NKT cells with certain SLAMF members, particularly Ly108, playing a major role therein [5,13,14,15,16,17,18,19,20,21].

Ly108 (SLAMF6, SF2000, NTB-A in humans) is mainly expressed on thymocytes, T cells, B cells, NKT cells, NK cells, and Dendritic Cells [19,22,23,24]. Ongoing research has resulted in an understanding that one major role of Ly108 in immunity is being present on the surface of specialized T cells and, through homotypic binding at cell interfaces, providing signals to developing B and NKT cells [19,25]. However, the specific mechanisms involved in this help have been shown to be more complex than previously thought. One example of this is the finding that the NKT deficiency and B cell defects in SAP-deficient mice could be reversed when crossed with Ly108 knockout mice [20]. It was also shown that SLAMF receptors supported NKT cell development by actually reducing the strength of the T cell signal, and that Ly108 could restore the NKT deficiency in a SLAMF knockout mouse [21].

Additionally, Ly108 plays a major role in cellular interfaces important for NK and cytotoxic T cells [26,27,28,29]. While perhaps expected of NK cells in the killing of hematopoietic target cells that express Ly108, cell surface interactions between NK cells and hematopoietic cells are also important for killing of non-hematopoietic cells (that do not express Ly108) [29]. Ly108/NTB-A in humans is extremely important for NK cell-mediated cytotoxicity [30] and B cell maturation [31], the latter also being dependent on signals provided by NTB-A on T cells [32]. At least in the case of NK cells, Ly108 provides an activating signal in the presence and binding of SAP (and probably EAT-2), and an inhibitory signal in their absence through binding of SHP-1 and SHIP-1 [8,29,33]. This complexity perhaps explains seemingly contradicting findings and presents a challenge moving forward. One important and potentially contributing factor that needs to be considered is that *Ly108* has four splice isoforms that are variably expressed and have different intracellular tails [22,34,35,36,37].

The *Ly108* gene is found within a region on chromosome 1 containing the nine members of the SLAM family [4]. This region has been implicated in congenic models of murine Lupus, as well as in the development of SLE in humans [38,39,40]. Significant differences in expression and sequences of SLAM family members are found at this locus in the mouse and can be divided into two haplotypes. These differences contribute towards the loss of tolerance as autoimmunity develops when the haplotype-2 SLAM family locus derived from NZW or 129 is present on the C57BL/6 (B6) background, which normally has a haplotype-1 SLAM locus [38]. One significant difference found between strains was in the level of mRNA expression of the two original isoforms of *Ly108*, with more of the *Ly108-1* isoform and less of the *Ly108-2* isoform in the Lupus-prone Sle1b mice [34]. Subsequent experiments showed that Ly108-2 could sensitize immature B cells to deletion, thereby possibly preventing autoimmunity by the negative selection of autoreactive cells [41]. Following this, Ly108-3 was described and although expression levels in primary cells have not been shown, it also has two ITSMs like Ly108-1 and Ly108-2, with signaling potential (phosphorylation) intermediate between these isoforms [22]. Alternative splicing of *Ly108/SLAMF6* is also present in humans, and although the splicing affects the extracellular domains, isoforms functioned differently [42].

Using protein detection, we previously identified Ly108-H1, a fourth isoform that was notably absent in two congenic mouse models of Lupus, with one other striking difference; it has only one ITSM [35]. Importantly, introduction of Ly108-H1 expression into Sle1b mice ameliorated Lupus-like autoimmunity [35]. Analysis of signaling has shown that, in contrast to Ly108-1 and Ly108-2, tyrosine phosphorylation Ly108-H1 was not detected. As there was also little SAP binding, it was suggested that this isoform could function as a decoy receptor [36]. Based on analysis using mutation of SLAM and NTB-A, however, we expected the SAP binding to the one ITSM present in Ly108-H1 to be preserved [9,43].

To determine the cellular and molecular basis of the suppressed immune response seen in animals expressing Ly108-H1, we used transfection of cell lines to perform functional and biochemical experiments. We were able to detect phosphorylation of Ly108-H1 as well as relatively normal SAP binding. We found, in contrast to Ly108-2, no evidence that Ly108-H1 was associated with activation-induced cell death (AICD), but rather suppressed cytokine production. After alignment of *Ly108* isoform sequences, we were able to define targets with which we were able to demonstrate the expression of Ly108-3 at the protein and mRNA level, while simultaneously comparing phosphorylation. 

## 2. Results

### 2.1. Alignment of Isoforms Illustrates Sequence Homology, but also Potential Signaling Differences

**Figure 1:**
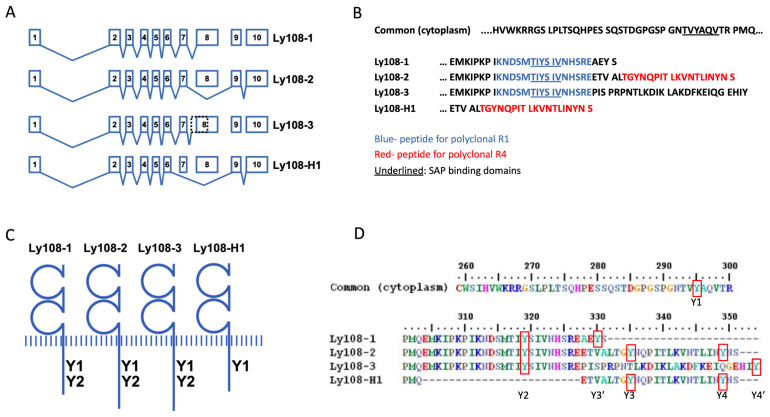
Schematic comparison of Ly108 isoforms. (**A**) Exon-intron organization of murine *Ly108* (*SLAMF6*) with alignment of *Ly108* isoforms. The *Ly108-2* isoform is generated by skipping exon 8 with transcription of exon 9 and 10. Ly108-3 is the result of an alternative splice acceptor site in intron 7 resulting in a frame shift. *Ly108-H1* is the result of skipping exon 7 and 8. (**B**) Alignment of amino acid sequences from the cytoplasmic domains of Ly108 showing SAP binding sites (underlined) and the sequences used to generate polyclonal antibodies R1 (blue) and R4 (red). (**C**) Illustration of Ly108 isoforms with the position of the tyrosines corresponding to ITSMs (SAP/EAT-2 binding sites) indicated. (**D**) Experimental ITSMs and plausible phosphorylation sites in the different Ly108 variants. Tyrosine residue annotation (red squares) using Ly108-2 as reference appear in position 295, 319, 335, and 349 (Y1–Y4). Other tyrosines shown are Y3′ in Ly108-1 and Y4′ in Ly108-3.

We performed sequence alignment of the four *Ly108* isoforms to determine if there were any significant differences that could explain the functional and biochemical differences previously reported between isoforms [22,35,36,41]. In Figure 1 we show the alternative splicing and exon use of the *Ly108* gene. *Ly108-1* and *Ly108-2* are the result of mutually exclusive exon splicing, *Ly108-3* has an alternative splice acceptor site proximal to exon 8, and *Ly108-H1* is the result of exons 7 and 8 being skipped. When mapping the SAP binding sites (underlined in Figure 1B) Ly108-H1 uniquely has only one ITSM, while the other isoforms have two. Also evident is that, while the mRNA sequences between *Ly108-1* and *Ly108-3* are very similar, Ly108-3 has a different and unique peptide sequence in its tail due to a frame shift [22].

Searching the PhosphoSitePlus database with the tails of Ly108 isoforms showed, in addition to the described ITSMs, various tyrosines assigned as putative phosphorylation sites [44]. Additional differences in protein binding motifs are shown in Supplementary Figure S1. When comparing these predictions, it remains apparent that the most striking difference with regards to phosphorylation resides in the absence of the second ITSM in Ly108-H1. For this reason, we proceeded to compare Ly108-H1 with this previously studied Ly108-1 and Ly108-2.

### 2.2. Ly108-H1 Binds SAP Regardless of Low Levels of Phosphorylation

**Figure 2:**
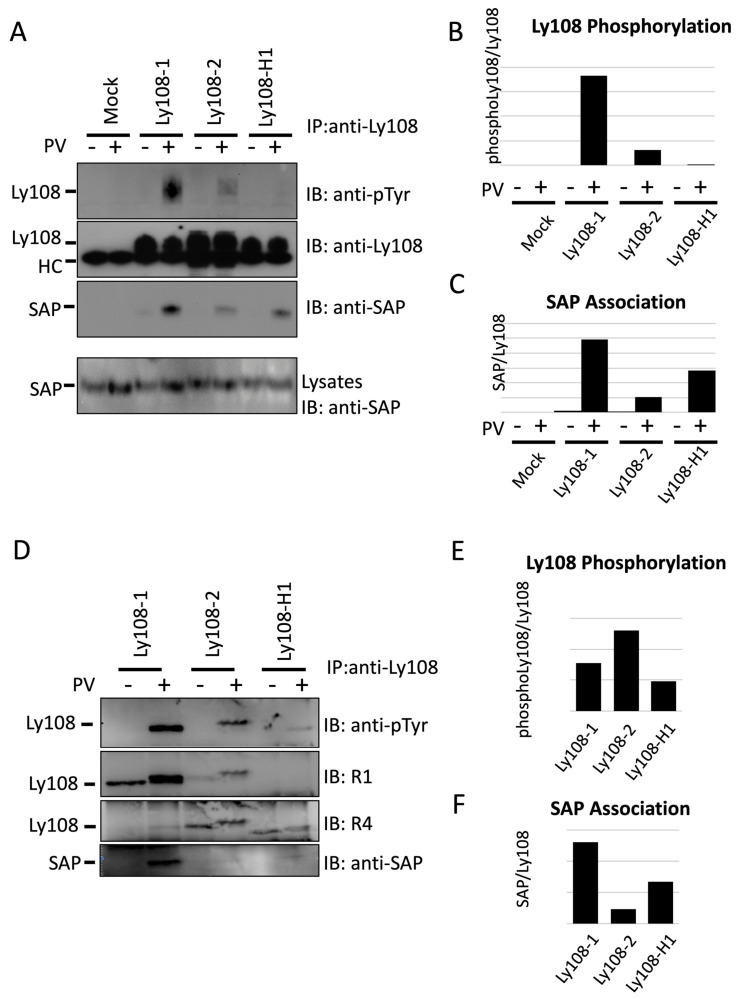
Differential phosphorylation and SAP binding by Ly108 isoforms. (**A**) Induction of Ly108 phosphorylation and SAP binding with pervanadate (PV) treatment of BI-141 cells expressing individual isoforms. Ly108 isoforms were immunoprecipitated and probed with anti-pTyr (**top** panel). The amount of total Ly108 was determined by re-probing with mouse anti-Ly108 mAb 13G3 (**upper middle** panel). The Ly108-SAP association was determined by probing the Ly108 immunoprecipitates with anti-SAP (**lower middle** panel). Levels of SAP in whole lysates were determined by probing with ant-SAP (**bottom** panel). Results are representative of three experiments (**B**) Phosphorylated of Ly108 was quantified and shown relative to total Ly108. Results of quantification are expressed as arbitrary units. (**C**) SAP association quantified relative to total Ly108. Results of quantification are expressed as arbitrary units. (**D**) Improved detection of phosphorylated Ly108-H1. Pervanadate treatment, immunoprecipitation, and immunoblotting were performed as above but precipitates were subjected to deglycosylation with PNGase-F in an intermediary step. Note that the gel shift due to phosphorylation is now more evident. A single experiment was performed. (**E**) Quantification of phosphorylation of Ly108 is shown relative to total Ly108. Total Ly108 was estimated with R1 and R4 by using Ly108-2 to normalize obtained values from pervanadate-treated lanes. (**F**) SAP association was quantified relative to total Ly108 in the pervanadate-treated lanes. Results of quantification are expressed as arbitrary units.

SAP binding to Ly108 is important for its signaling and has been shown to be dependent on phosphorylation [22,36]. We selected Ly108-H1 to examine tyrosine phosphorylation and SAP binding because both may be influenced by its unique characteristic of only having one ITSM. We expected phosphorylation and phosphorylation-dependent SAP binding based on previous studies that utilized targeted mutations of ITSMs to characterize SLAMF1, NTB-A, and Ly108 [43,45,46]. Using the BI-141 T-cell hybridoma transfected with SAP and individual Ly108 isoforms we confirmed that Ly108-2 is less phosphorylated than Ly108-1, as shown in Figure 2A,B [22,36]. Similarly to Dutta et al. we saw almost no detectable phosphorylation of Ly108-H1, with a minimal signal shown using quantitation software (last column Figure 2B) [36]. In contrast to their findings, we show in Figure 2A,C that Ly108-H1 can effectively bind SAP after treatment with the phosphatase inhibitor pervanadate. This prompted us to question whether phosphorylated Ly108-H1 had escaped detection because of dilution in the naturally occurring smeared band of the glycosylated form of the protein. To address this, we performed separate experiments to concentrate the immunoprecipitated Ly108 isoforms by deglycosylation with PNGaseF. This resulted in clear bands and detectable phosphorylation of Ly108-H1, as seen in Figure 2D (top panel). Quantification of relative phosphorylation proved difficult as the pan anti-Ly108 monoclonal antibody 13G3-19D does not detect deglycosylated Ly108, and neither polyclonal detects all isoforms. To estimate total Ly108 we used the pervanadate-treated Ly108-2 to normalize the R1 and R4 results. With this method, we see Ly108-2 to be slightly more phosphorylated than Ly108-1 and Ly108-H1, but these results may be further confounded as R1 unexpectedly showed a higher signal in pervanadate-treated lanes. SAP binding to the three isoforms was confirmed and the levels shown in Figure 2D (bottom panel) and Figure 2F are consistent with those seen in the previous experiment. SAP binding to Ly108-H1 could also be seen in thymocytes from transgenic mice (Supplementary Figure S2).

These results show, therefore, that Ly108-H1 is phosphorylated after which it effectively binds SAP, even though detection of Ly108 phosphorylation was challenging.

### 2.3. Suppression of Cytokine Production by Ly108-H1

To define the role of Ly108-H1 in T cell function, we measured cytokine production in BI-141 clones that were transfected to stably-expressed SAP and individual isoforms, in a manner previously described [45,47]. In line with phosphorylation experiments, we compared clones expressing Ly108-H1 to those with Ly108-1 and Ly108-2, as well as clones lacking Ly108 (Mock).

**Figure 3:**
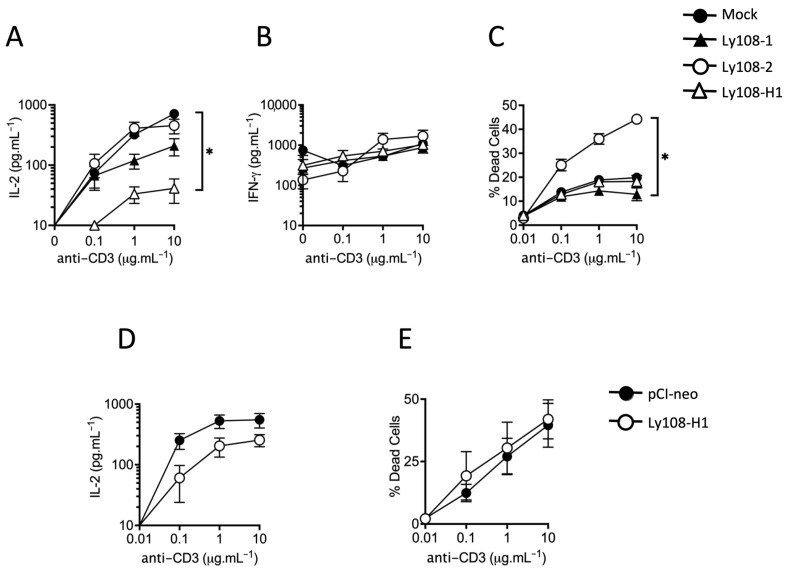
Suppression of IL-2 production by Ly108-H1 in T cell lines. (**A**,**B**) Individual clones of BI-141 cells stably expressing SAP and individual isoforms were stimulated with the indicated concentrations of plate-bound anti-CD3. After 20 h IL-2 (**A**) or (**B**) IFN-gamma was determined by ELISA. Data points indicate mean ± SEM of three separate clones (Mock: A1, A2, A3. Ly108-1: A1, A5, D4. Ly108-2: C1, C4, C6. Ly108-H1: A7, A17, A18). Each clone was assayed in triplicate. Similar results were obtained in three separate experiments. We performed a Kruskal-Wallis test resulting in *p* < 0.0001. Shown is the Dunn’s post-test significance comparing individual columns: * *p* < 0.05. Note the logarithmic scale. (**C**) AICD was determined by FACS in BI-141 cells stimulated with anti-CD3 using DAPI as a marker for dead cells. Each clone was assayed singularly with data from 10,000 cells collected. Data points indicate mean ± SEM of three separate clones (Mock: A1, A2, A3. Ly108-1: A1, E4, E6. Ly108-2: A1, C1, C4. Ly108-3: A17, A18, B6). Similar results were obtained in two separate experiments. (**D**) IL-2 production in DO11.10 cells stably expressing Ly108-H1 or the empty vector pCI-neo were performed as above. Data points indicate mean ± SEM of four separate clones (control pCI-neo: A1, A2, A4, A5. Ly108-H1: A3, A4, A11, A12). Each clone was assayed in triplicate. Similar results were obtained in four separate experiments. (**E**) AICD in DO11.10 cells stably expressing Ly108-H1 or the empty vector pCI-neo was performed as above. Each clone was assayed singularly with data from 10,000 cells collected. Data points indicate mean ± SEM of 5 separate clones (control pCI-neo: A1, A2, A3, A4, A5. Ly108-H1: A3, A4, A8, A11, A12). Similar results were obtained in two separate experiments.

The stimulation of BI-141 clones expressing SAP and Ly108 resulted in less IL-2 production than those expressing SAP alone. We consistently saw that Ly108-H1 expression resulted in the greatest reduction of IL-2 production (Figure 3A). A less pronounced reduction in IL-2 was also observed with Ly108-1 expressing clones when compared to control and Ly108-2 expressing clones, although we cannot exclude that this result may be influenced by lower levels of Ly108 on the transfected cells (Supplementary Figure S3). Expression of the individual isoforms of Ly108 had no notable effect on IFN-γ production, as shown in Figure 3B. We ruled out reduced IL-2 being due to excessive activation but did see increased AICD with Ly108-2 as seen in Figure 3C. To examine IL-2 production and AICD in another Ly108-negative T-cell line, we generated stably-transfected DO11.10 T-T hybridoma cells lines expressing Ly108-H1. Here, too, we saw some inhibition of IL-2 production by Ly108-H1, albeit to a lesser extent than that seen in BI-141 cells, when compared to empty vector control (Figure 3D). As in the BI-141 cells, Ly108-H1 did not enhance AICD in DO11.10 cells, as shown in Figure 3E.

In addition to these findings, we performed limited experiments to analyze the effect of individual isoforms on apoptosis using immature thymocytes and the WEHI-231 cell line as described for the two original Ly108 isoforms [41]. Here, we observed that overexpression of Ly108-2 resulted in more apoptosis than Ly108-1, while Ly108-H1 did not show a clear pattern of enhancement (Supplementary Figure S4).

In summary, our findings support those of Kumar and colleagues for Ly108-2 regarding AICD, while demonstrating that there does not seem to be a role for Ly108-H1 therein [41]. Expression of Ly108-H1, and to a lesser extent Ly108-1 and Ly108-2, did, however, result in suppressed IL-2 production in T cell lines.

### 2.4. Detection of Phosphorylated Isoforms, including Ly108-3, in Primary Cells

**Figure 4:**
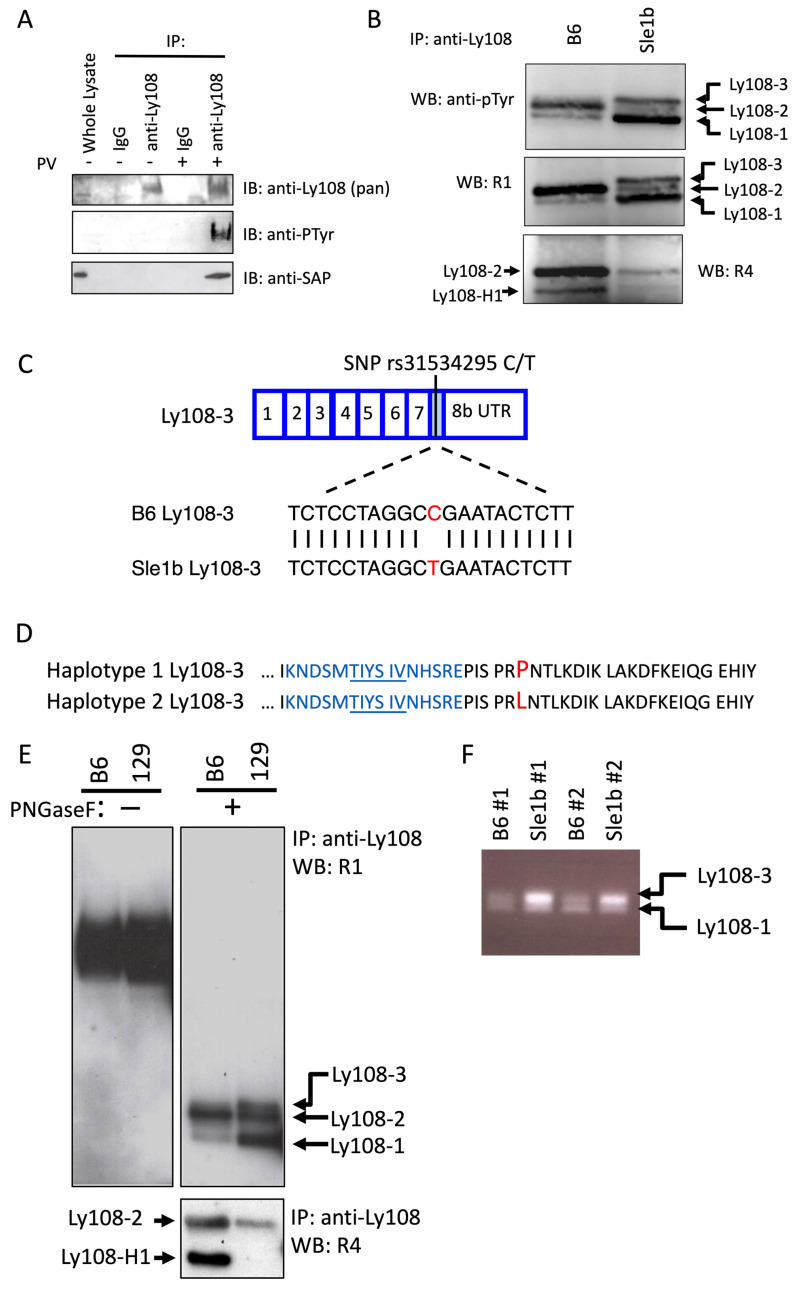
Ly108 isoforms, including Ly108-3, are differentially expressed, and phosphorylated in primary cells. (**A**) Suspended thymocytes from B6 mice were treated where indicated for 20 min with pervanadate (PV+) before lysis, after which immunoprecipitation was performed with the indicated antibodies or isotype control (IgG). After SDS-PAGE and transfer, membranes were blotted for Ly108 and SAP. Phosphorylated Ly108 was detected on stripped membranes that were re-probed with anti-phosphotyrosine. (**B**) Thymocytes from the indicated strains were treated with pervanadate before lysis. Ly108 was immunoprecipitated (IP) and deglycosylated. After separation by SDS-PAGE and transfer to membrane, phosphorylated isoforms were detected by anti-phosphotyrosine antibody by Western blotting (WB). Further re-probing was performed with polyclonals R1 and R4 antibody to demonstrate specificity. Individual isoforms are indicated with the help of arrows. (**C**) Exon organization of *Ly108-3* showing the location of the non-synonymous SNP rs31534295 (in red) and alignment of a short portion of Sle1b derived *Ly108-3* with B6. Shown is the SNP and 10bp flanking sequence. (**D**) The resulting amino acid substitution (in red) in the tail of Ly108-3 is shown in relation to the second SAP binding site (underlined) and target of polyclonal antibody R1 (blue). (**E**) Immunoprecipitation of Ly108 from unstimulated B cells from the indicated mouse strains. Some samples were deglycosylated (in the **right** panels) and Western blotting was performed with the polyclonal antibodies R1 (**top** panels) or R4 (**bottom**). Elbow arrow directed at a band corresponding to Ly108-3. (**F**) Agarose gel (1–3%) electrophoresis. For *Ly108-1* and *Ly108-3* expression in thymus RT-PCR products were amplified using oligonucleotides covering a region between exon 7–8. Bands of 227 bp and 241 bp correspond to *Ly108-1* and *Ly108-3*. Two mice from each strain are shown.

To confirm that SAP does not bind unphosphorylated Ly108 in primary cells we established co-immunoprecipitation conditions with cell suspensions of murine thymocytes, as Ly108 in whole thymus is phosphorylated [36]. As shown in Figure 4A, using a monoclonal antibody (13G3) that binds all four isoforms, we were unable to co-immunoprecipitate SAP unless the cells were treated with pervanadate. Due to the dependance of SAP binding on Ly108 phosphorylation we set out to compare phosphorylation of Ly108 isoforms in primary cells.

We took two approaches to compare phosphorylation as well as detect Ly108-3. First, based on previous findings and predictions, we knew that Ly108-3 could be phosphorylated and, therefore, detected using an anti-phosphotyrosine antibody [22]. Second, alignment of the amino acid sequences, together with the targets of our previously generated polyclonal antibodies shown in Figure 1B, lead us to believe that Ly108-3 should also be detectable with antibody R1 (Figure 1B, target shown in blue). R1 had initially been generated to detect Ly108-1 and Ly108-2, but Ly108-3 contains an identical target sequence and is very similar in molecular weight to Ly108-2 (39.1 vs. 38.6 kDa, respectively). Both strategies required pervanadate treatment, as well as deglycosylation and additional separation of isoforms by SDS-PAGE. In the top panel of Figure 4B, three bands of phosphorylated Ly108 are visible in the lane from Sle1b mice. Three corresponding bands are also visible in the middle panel of membranes re-probed with R1 and correspond with Ly108-1, Ly108-2, and Ly108-3, as indicated. As expected, re-probing with R4 in the lower panel resulted in only one band (Ly108-2) in Sle1b mice and two bands (Ly108-H1 and Ly108-2) in B6 mice. Consistent with previous studies, Ly108-1 is the most heavily phosphorylated, followed by Ly108-3 and then Ly108-2 [22,36]. Ly108-H1 phosphorylation in this experiment is not clearly seen (Figure 4B, top panel), but barely visible on the original films, which is also consistent with previous findings [36].

While it seems that Ly108-3 is preferentially expressed in Sle1b mice at the protein level, the experimental conditions are not suitable for accurate comparison. Interestingly, in our search for an explanation for differences in expression we became aware of a non-synonymous single nucleotide polymorphism (SNP) in *Ly108-3*. Shown in Figure 4C is the site of the SNP and the resulting alignment with B6 after sequencing *Ly108-3* from Sle1b mice. We searched a database comparing mouse strains and found that SNP variant present in Sle1b mice is shared with other SLAM haplotype-2 mice [48]. This polymorphism results in a proline to leucine substitution just downstream of the second ITSM and target of polyclonal R1, as shown in Figure 4D.

To test sensitivity of the R1 antibody for Ly108-3 from B6 mice and address potential conformational changes due to this SNP, we used immunoprecipitated B cell lysates that had not been treated with pervanadate, as this has been reported to reduce Ly108 expression [36]. We also compared B6 to 129 wild-type mice to see if Ly108-3 expression was associated with SLAM haplotype-2. Here, too, we could see a discrete band of a protein slightly larger than Ly108-2 (Figure 4E elbow arrow) in 129 mice that is absent when re-probed with R4 (Figure 4E lower panel). This detection pattern is in agreement with that expected of Ly108-3 and confirms that R1 also recognizes unphosphorylated Ly108-3. The increased expression of Ly108-3 protein seems, therefore, to be common in haplotype-2 mice.

Because the differential Ly108-3 expression had only been partly addressed, we performed a semi-quantitative RT-PCR on cDNA from B6 and Sle1b mice using primers that amplify *Ly108-1* and *Ly108-3* with amplicons of slightly different lengths. As seen in Figure 4F we do see *Ly108-3* mRNA present in B6 mice, although it is more prominent in Sle1b.

## 3. Discussion

SAP-deficient humans and mice have impaired NK and T cell mediated cytotoxicity and have a major defect in humoral immunity [1,2,4,49]. These processes require positive or negative signals which can be supplied by Ly108 (SLAMF6) through homophilic cell surface interactions. The resulting tyrosine phosphorylation of ITSMs allows for binding of the adaptor SAP, or the competing molecules SHP-1 and SHIP-1, to Ly108′s cytoplasmic tail [8]. Importantly, due to alternative splicing, Ly108 has four isoforms with differing cytoplasmic tails, three of which are differentially expressed in Lupus-prone mouse strains [34,35].

The ability to bind the adapter proteins SAP is of utmost importance to the function of those SLAMF members with ITSMs [4,8]. A comparison of binding motifs in Ly108 isoforms shows that Ly108-H1 is unique in that it has one ITSM, while others have two. Previously, analysis of Ly108-H1 showed no tyrosine phosphorylation and little SAP binding [36]. However, we were able to detect low levels of phosphorylated Ly108-H1 after treatment with pervanadate, as well as SAP binding. We think the phosphorylation of Ly108-H1 seen represents the tyrosine contained within the first ITSM because SAP binding was phosphorylation dependent. We also propose that the second ITSM is crucial for further signaling as was shown with SLAMF1, where SAP binding resulted in Fyn-T recruitment, and phosphorylation of distally located tyrosines [45,50]. In addition, it was shown that the second ITSM of SLAM was crucial for signaling through phosphorylation of the downstream mediators Dok-1, Dok-2, and SHIP-1 [45]. The second ITSM is also essential for the function of NTB-A in NK cells [43]. We propose, therefore, that differences between downstream signaling will be more pronounced when comparing Ly108-H1 to other isoforms because it lacks a second ITSM but does bind SAP.

Previous work supported a protective role for Ly108-2 in the protection against Lupus-like autoimmunity in mice by augmented B cell receptor signaling, possibly resulting in a lower threshold for self-reactive immature B cells to undergo negative selection [41]. We later demonstrated that introduction of a *Ly108-H1* transgene into Lupus-susceptible congenic mice could ameliorate disease and dampen T and B cell activation [35]. A similar effect could also be attained by transferring T cells expressing Ly108-H1 and here we show that Ly108-H1 had a pronounced inhibitory effect on IL-2; thus, providing support for a T cell mediated regulation of inflammation.

In the context of mouse models of Lupus, expression patterns of Ly108-1, Ly108-2, and Ly108-H1 have been measured in primary cells at the protein and mRNA level from wild-type and Sle1b mice [35,36]. Because little is known of Ly108-3, we set out to detect it at the protein level. We found Ly108-3 protein expressed in thymocytes and B cells from Sle1b mice using extensive separation of isoforms after phosphorylation, immunoprecipitation, and Western blotting with anti-phosphotyrosine antibodies. Expression was confirmed using an antiserum that was predicted to detect Ly108-3, and probably not previously seen due to insufficient separation of isoforms [35]. We could not clearly detect Ly108-3 protein in B6 lysates, and although only assessed in a semi-quantitative manner, we do see preferential mRNA expression of *Ly108-3* in Sle1b mice. While trying to explain these differences in Ly108-3 expression we became aware of a non-synonymous SNP which could be of importance. Examination of the primary structure of Ly108-3 shows this SNP located just distally from a SH3 binding site and the target of the polyclonal antibody (Figure 1 and Figure S5). The version of SNP translates into proline in B6 mice, or a leucine in Sle1b and other haplotype-2 mice. It is possible that the proline results in a conformation that is more amenable for binding of adaptor proteins, something that would be of great interest to attain. Therefore, Ly108-3 protein expression, as well as the variant of SNP, seems to distinguish SLAM haplotypes.

Quantification of protein isoforms is of great importance but can be challenging. For example, we previously showed *Ly108-1* mRNA levels to be comparable between B6, 129, and Sle1b mice when we performed by RT-PCR [35]. This was not in agreement with quantitative PCR data from other studies showing higher levels of *Ly108-1* in 129 and Sle1b mice [34,36,41]. An explanation for this discrepancy lies in sequences shared between *Ly108-1* and *Ly108-3* isoforms. Using oligonucleotides with targets common to both isoforms would have resulted in amplification of both during quantification, and *Ly108-3* seems to have higher expression in 129 and Sle1b mice. As previously noted, this was the case for *Ly108-2* where mRNA levels initially measured would have been overestimated because of the then-unknown sequence homology with *Ly108-H1* [36], and may have also been the case with protein levels [35]. Taken together, previous mRNA and protein detection including that done in our own laboratory would have inadvertently been inaccurate due to non-specific cross-reactivity of oligonucleotides and antibodies because of isoform homology [34,35]. While Ly108-H1 and Ly108-3 expression seems predominately regulated at the genomic level, accurate expression levels between cell types at various stages of development of activation has yet to be attained.

Ly108-H1 molecules may regulate Ly108 signaling by retaining the capability to bind their extracellular ligands, i.e., all Ly108 isoforms, as well as recruiting the shared intracellular ligand SAP. The capability to signal through downstream mediators may be lacking due to there being only one ITSM and would mean that Ly108-H1 would be competing at two levels with the other Ly108 isoforms. In addition, we show differential expression of Ly108-3, an isoform with potentially interesting signaling properties. We propose that Ly108-H1 and Ly108-3 may be more important than the other isoforms in the context of murine SLE for three reasons. First, the strain-specific differences of Ly108-1 and Ly108-2 do not seem as robust as that of Ly108-H1 and Ly108-3. Second, Ly108-H1 has been the only isoform shown to protect against murine SLE *in vivo*, although there was still some residual disease [35]. Third, a non-synonymous SNP could affect signaling due to the structural change it provides in proximity to binding domains, thereby also justifying that Ly108-3 is a clear candidate for further characterization. The recent demonstration that alternative splicing of SLAMF6 in humans could be steered by the use of antisense oligonucleotides, thereby increasing anti-tumor effect of tumor infiltrating lymphocytes, is further evidence of the importance of this field of study [42].

Thus, the current work supports the importance of increased awareness of isoforms as these are more abundant than previously thought and can confound expression data [51,52,53,54,55]. We provide additional evidence that isoforms have varying functions which may be important for the resolution of the inflammatory response and could provide insights into additional lines of drug development as well as preventing off-target adverse effects [56].

## 4. Materials and Methods

### 4.1. Expression Vectors

*Ly108* isoforms were amplified by PCR with primers introducing XhoI and XbaI restriction sites and cloned into pCR2.1-TOPO (Invitrogen, Grand Island, NY, USA) before subcloning into the mammalian expression vector PCI-neo (Promega, Madison, WI, USA). *Ly108-1* and *Ly108-2* templates were kindly provided by Dr E. Ruley. *Ly108-H1* was amplified from C57BL/6 thymus.

### 4.2. Transfection

BI-141 cells were maintained in complete media (RPMI 10% Fetal Calf Serum) supplemented with glutamine and penicillin/streptomycine. Stable SAP transfectants were obtained by transfection of an SAP IRES GFP retroviral vector followed by cell sorting. For transfection of *Ly108* isoforms, 1–2 × 10^7^ BI-141 cells were transfected by electroporation (250 V, 960 μF) with 10 μg of plasmid DNA in 400 μL OptiMEM (Gibco, Grand Island, NY, USA) using a cuvette with a 4 mm electrode gap (Bio-Rad, Richmond, CA, USA). When generating stable transfectants, cells with surface expression were sorted by FACS using the FACSAria (BD Biosciences, San Jose, CA, USA) after 48 h and maintained in media containing the selection antibiotic G418 (0.6 mg/mL) for one week before a second round of cell sorting. Individual clones were isolated by limiting dilution in selection media. DO11.10 cells (kindly provided by Dr J. Buhlman) were transfected by AMAXA technology using the manufacturers protocol for EL-4 cells.

### 4.3. Mice

C57BL/6 (B6) mice were purchased from Jackson (Bar Harbour, ME) and 129SvEvTac (129) mice from Taconic (Hudson, NY, USA). B6.Sle1b (Sle1b) mice were kindly provided by Dr L. Morel. All animals were housed in the animal facility of the Beth Israel Deaconess Medical Center.

### 4.4. Cell Stimulation

For cytokine production and activation-induced cell death BI-141 and D011.10 cells were stimulated with the indicated amounts of plate bound anti-CD3 (2C11) for 20 h in complete media. Cytokine levels were determined by ELISA as per the manufacturers’ instructions (BD Pharmingen, San Diego, CA, USA). Dead cells were determined by DAPI (0.5 μg/mL) uptake using FACS analysis.

### 4.5. Immunoprecipitation and Western Blotting

To phosphorylate Ly108 and perform co-immunoprecipitations assays, cells were treated with pervanadate for 10 min before lysis. Lysed cells were solubilized with the detergent Brij 98. Ly108 was precipitated from lysates with anti-Ly108 (13G3-19D) and protein-G agarose (Invitrogen) followed by denaturing in glycoprotein denaturing buffer. Proteins were separated on a 4–12% gradient SDS-PAGE gel with MOPS running buffer (Invitrogen). After transfer to PVDF membrane, Western blotting was performed with the indicated antibody. Phosphotyrosine was detected by Western blot using the monoclonal antibody 4G10 (Upstate, Lake Placid, NY, USA). These were followed by species-specific secondary HRP-conjugated antibodies (Jackson ImmunoResearch, West Grove, PA, USA). Reactivity was detected by chemiluminescence with Supersignal (Pierce, Rockford, IL, USA).

Individual isoforms were separated as previously described [35]. Briefly, immunoprecipitated Ly108 was resolubilized before deglycosylation with PNGaseF and separated by SDS-PAGE gel with MOPS running buffer. Transfer and detection were performed as above but using the polyclonal antibodies R1 and R4 as described before [35]. Quantification was performed with ImageJ.

### 4.6. RT-PCR and Sequencing

RNA was extracted from cells using the RNeasy kit (QIAGEN, Valencia, CA, USA) or TRIZOL (Invitrogen). A shorter RT-PCR of *Ly108-3* and *Ly108-1* was performed with forward primer: 5′-TCATTCCAGAGAGCCCATTT-3′ and reverse primer 5′-GAAGGATCCAGGCTGAAGTG-3′. A full-length RT-PCR of *Ly108-1* and *Ly108-3* was performed on cDNA templates with the following primers with introduction of restriction sites: Ly108-start: 5′-GGCTCGAGATGGCTGTCTCAAGGGCT-3′; Ly108-1-end: 5′-GGTCTAGATTAAGAGTATTCG-GCCTCTCTGG-3′ before subcloning into vectors for sequencing. Sequences were determined at the Beth Israel Deaconess Medical Center sequence facility and nucleotide sequences were assembled and aligned using Vector NTI Advance (Invitrogen). Each RT was performed with the Protoscript cDNA kit (New England Biolabs, Ipswitch, MA, USA).

### 4.7. Sequence Alignment and Annotation

Alignment of Ly108 tail sequences was performed with the help of BLAST from the National Center for Biotechnology Information (NCBI) [57] and SnapGene software version 6.2.0 (www.snapgene.com).

The PhosphoSitePlus database was used to determine potential phosphorylation sites at www.phosphosite.org, accessed on 18 November 2022 [44].

### 4.8. Stastical Analysis

Multiple comparisons (BI-141 cells) were analyzed using the non-parametric Kruskall-Wallis test with the Dunn’s post-hoc test. Single comparisons (DO11.10) were performed using the Mann-Whitney test. Statistical analysis was performed using GraphPad Prism version 9.4.1 for macOS, GraphPad Software, San Diego, CA, USA, www.graphpad.com.

## Data Availability

Data is contained with the article.

## Supplementary Materials

The following supporting information can be downloaded at: https://www.mdpi.com/article/10.3390/ijms24055024/s1. References [58,59] are cited in the Supplementary Materials.
