# Supplementary material for: Characterization of Ly108-H1 Signaling Reveals Ly108-3 Expression and Additional Strain-Specific Differences in Lupus Prone Mice
Source: Int J Mol Sci. 2023 Mar 6;24(5):5024. doi: 10.3390/ijms24055024 (PMC10003074; doi:10.3390/ijms24055024)
Supplement: Supplementary file 1 [file ijms-24-05024-s001.zip › ijms-2193318-supplementary.pdf]

Supplementary Data

Supplementary legends and methods

Supplementary Figure S1.
Prediction results of eukaryotic linear motifs (ELM) searches using the cytoplasmic tails of individual isoforms at the ELM resource for Functional Sites in Proteins www.elm.eu.org

Supplementary Figure S2.
Ly108-H1 expressed as a transgene in Ly108-/- mice binds SAP. Ly108-1 and Ly108-H1 were cloned into the human CD2 cassette as previously described by Greaves *et al* Cell 1989. Removal of vector backbone was performed by digestion with KpnI and SalI prior to purification by agarose gel electrophoresis and dialysis. Purified DNA was injected into fertilized C57BL/6 oocytes. Screening of founders was performed by PCR and these crossed with Ly108 knockout mice. Thymocytes were treated with pervanadate and deglycosylation, immunoprecipitation and western blotting was performed as in Figure 2B.

Supplementary Figure S3.
Stable expression of Ly108 isoforms in BI-141 cells as shown by FACS. Ly108 was detected with Cy-5 labeled anti-Ly108 (clone 13G3-19D) shown in open histograms. A Cy5 labeled IgG2a isotype control was purchased from (Beckman Coulter) and staining shown in filled histograms. Data was acquired with a LSRII or FACSCanto cytometer with HTS option (BD Pharmingen) and analysed using FlowJo software (Treestar).

Supplementary Figure S4.
Induction of apoptosis in (A) double-positive thymocytes and (B) WEHI-231 cells.
Thymocytes from 129Sv mice were transfected with individual isoforms by electroporation as previously described by Bell *et al* Nat. Med. 2001. The following day cells were stimulated with 25ug/mL plate bound anti-CD3 and 5ug/mL soluble anti-CD28 (manufacturer) or anti-Ly108 (13G3) for 3 hours. WEHI-231 (ATCC, Rockville, MD) were transfected with individual isoforms by AMAXA technology according to the manufacturers protocol. After 24 hours cells were stimulated with anti-IgM (Jackson Immunoresearch) for 24 hours.
Apoptosis was determined by FACS analysis by using AnnexinV binding and DAPI exclusion. Values shown are relative to unstimulated cells mock transfected with empty vector.

Supplementary Figure S5
ELM predictions of searches using the indicated tail sequence of Ly108-3 and filtered for hits flanking the non-synonymous SNP. Binding motifs are shown and the amino acids corresponding to the non-synonymos SNP indicated in red.

Supplemental Figure S1.

Prediction results of eukaryotic linear motifs (ELM) searches using the cytoplasmic tails of individual isoforms.

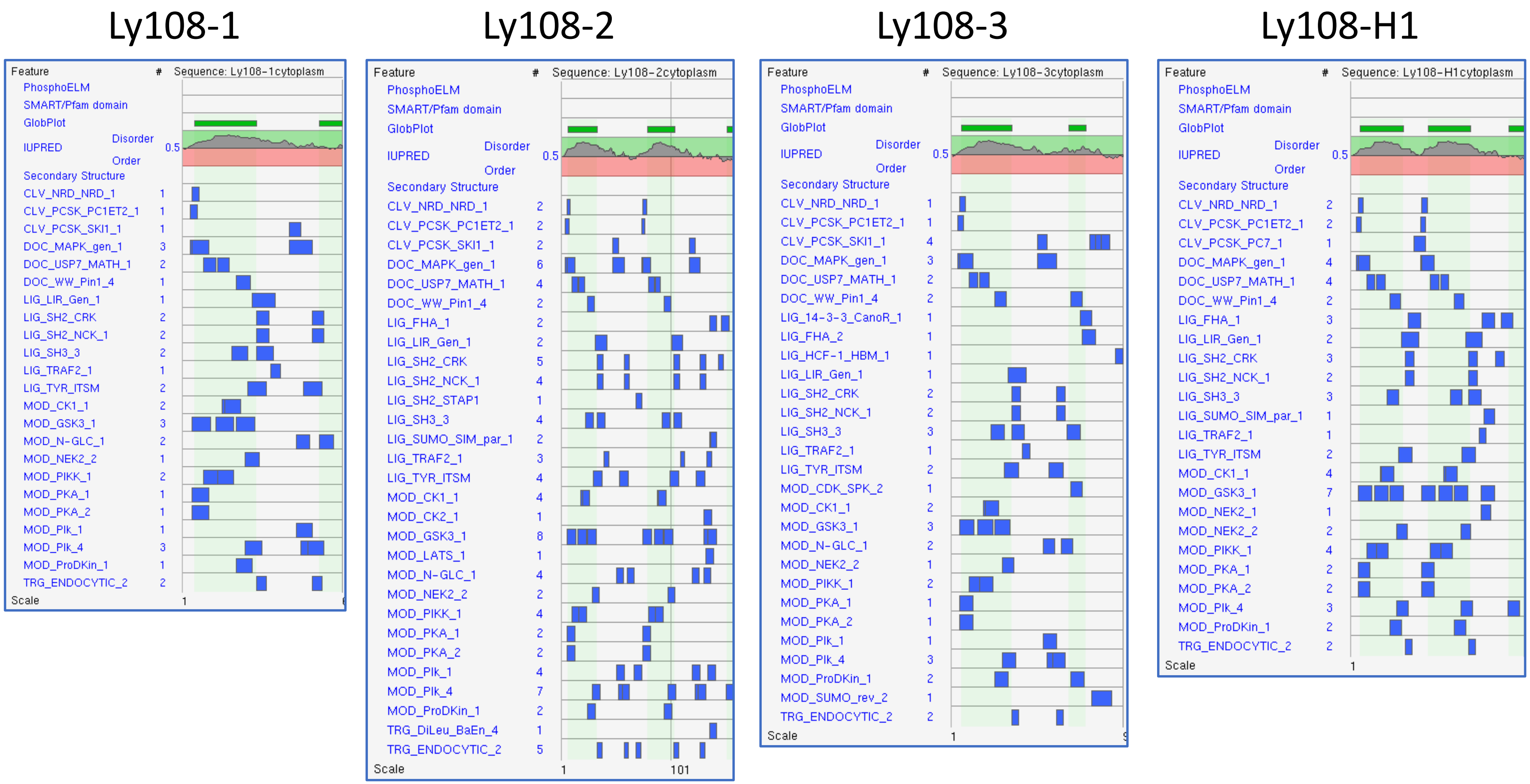

Supplemental Figure S2.
Ly108-H1 expressed as a transgene in Ly108-/- mice binds SAP

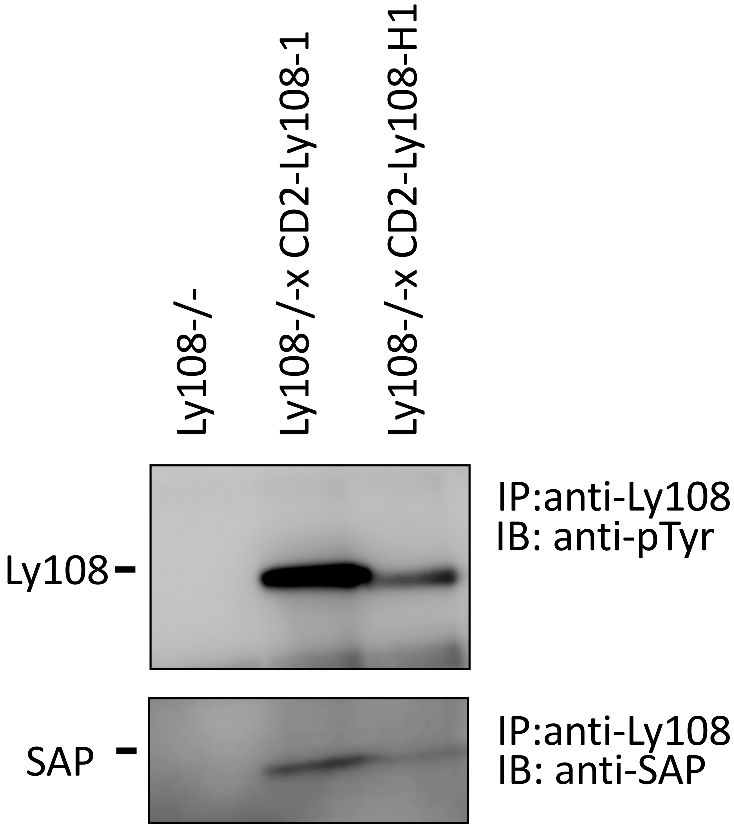

Supplemental Figure S3.
Stable expression of Ly108 isoforms in BI-141 cells as shown by FACS.

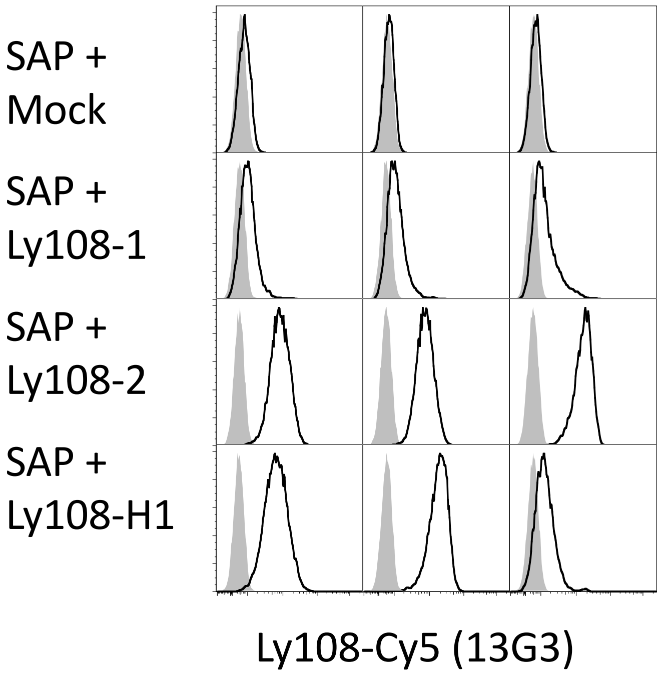

Supplementary Figure S4.
Induction of apoptosis in (A) double-positive thymocytes and (B) WEHI-231 cell

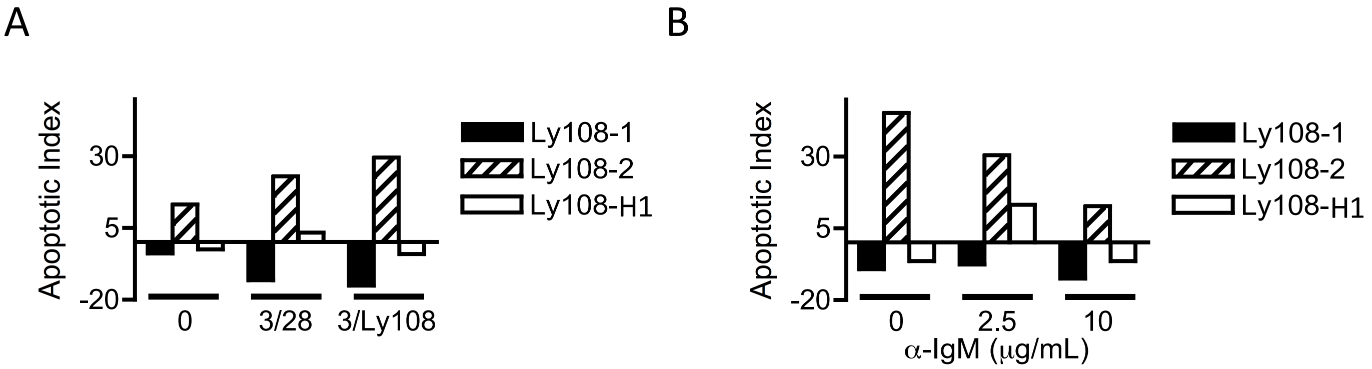

Supplementary Figure S5
ELM results filtered for sites flanking the non-synonymous SNP

Mouse	Query sequence
B6	… EMKIPKPIKNDSMTIYSIVNHSREPISPRPNTLKDIKLAKDFKEIQGEHIY
Sle1b	… EMKIPKPIKNDSMTIYSIVNHSREPISPRLNTLKDIKLAKDFKEIQGEHIY

ELM Code of site	Sequence of binding site	
LIG_SH3_3	SREPISP	
MOD_CDK_SPK_2	EPISPR	
DOC_WW_Pin1_4	EPISPR	
MOD_ProDKin_1	EPISPRP	(or EPISPRL)
LIG_FHA_2	PNTLKDI	(or LNTLKDI)
LIG_14-3-3_CanoR_1	RPNTLK	(or RLNTLK)
